# A fluorescence immunoassay for a rapid detection of *Listeria monocytogenes* on working surfaces

**DOI:** 10.1038/s41598-020-77747-y

**Published:** 2020-12-10

**Authors:** Alessandro Capo, Sabato D’Auria, Monique Lacroix

**Affiliations:** 1 INRS Armand-Frappier Health and Biotechnology Centre, Research Laboratories in Sciences, Applied to Food, Institute of Nutraceutical and Functional Foods (INAF), 531 Boulevard des Prairies, Laval, QC H7V 1B7 Canada; 2 Institute of Food Science (ISA), National Research Council, Laboratory for Molecular Sensing, Via Roma 64, 83100 Avellino, Italy

**Keywords:** Biomaterials, Biotechnology, Assay systems, Fluorescence spectroscopy, Immunological techniques

## Abstract

*Listeria monocytogenes* is a foodborne pathogen responsible for human listeriosis. The increasing incidence of listeriosis induced governments and food manufacturing enterprises to act to diminish the problem. Several methods for the detection of *Listeria monocytogenes* in food industries were developed. However, they are time-consuming and require the use of specialized equipment. To reduce the detection time of *Listeria monocytogenes* in food, in this work we developed a fluorescence sandwich immunoassay based on the use of an innovative chitosan-cellulose nanocrystal (CNC) membrane that improves the antigen capture during bacterial growth. The combined use of CNC film for the capture of p60 protein-specific antigen together with the use of fluorescence detection reduced the time of analysis from 24 to 12 h with a limit of detection (LOD) of the assay of 10^2^ CFU/mL (2 Log). In addition, the use of monoclonal anti-PepD covalently immobilized to a CNC membrane assured a high specificity of the assay. Interestingly, the obtained results show no cross-reactivity with the five most diffused pathogen bacteria strains tested.

## Introduction

*Listeria monocytogenes* (LM) is a remarkable foodborne pathogen in the world^1^ that can cause serious aggressive disease (listeriosis) in humans and other animal species^2^.

Listeriosis can occur in healthy individuals and in young, elderly, or immunocompromised individuals. Listeriosis manifests as septicemia, meningitis and mild gastroenteritis with a fatality rate ranges from 20 to 30%^3^.

Strains of LM can grow on floors, drains, surfaces and equipment within food industry premises, even if they are routinely cleaned and disinfected^4^. In Møretrø et al.^5^, twenty-one studies were mentioned in which the persistence of LM strains was demonstrated.

The presence of LM has been demonstrated in many raw and processed food products^6^. It has been also identified in environmental samples at retail establishments^7,8^ and at home of consumers^9,10^.

The principal bacterium access to the organism is represented by digestive system. The approximate infection dose of LM is between 10^7^ and 10^9^ (CFU/gr) in healthy hosts and between 10^5^ and 10^7^ (CFU/gr) in subjects at risk of infection^11–13^.

To infect efficiently cells, LM acts with different virulence effectors on cellular infection cycle^14^. These effectors are proteins located at the surface of the bacterial cell or secreted to the extracellular environment. The p60 protein (p60) is a surface cell wall protein of *Listeria* species and it is released in the medium of culture^15,16^. It is responsible for the successful invasion of host cells^17,18^. This protein is secreted by all LM species. It has been shown that it exhibits a peptidoglycan (murein) hydrolase activity that is required for normal cell division^19,20^. Further studies have demonstrated that p60 is necessary for invasiveness^15,21^ and it play an immunomodulatory factor, owing to the generation of muramyl peptides from murein during infection in vivo^22^.

The p60 protein is a specific antigen used for detection of LM due to the presence of a short and unique hydrophilic peptide (QQQTAPKAPTE) called PepD^16,17,23,24^. All these features make this protein a perfect antigen for the specific detection of LM by a monoclonal antibody produced (anti-PepD mAb)^16,17^.

Two independent risk assessment studies in United States^25,26^ highlighted that 50% of human listeriosis cases were associated with consumption of ready-to-eat (RTE) deli meats contaminated at retail level. In Canada, a listeriosis outbreak was linked to contaminated cheese made from pasteurized milk^27^.

In 2008, a great listeriosis outbreak occurred in Canada. Consequently, as reported in FSnet archives (http://www.archives.foodsafety.ksu.edu/search.html), it was registered an increasing number of food withdrawals in Canada and in USA. Recently, the incidence of sporadic cases has risen in Europe Union (EU)^28–32^. The European Food Safety Authority (EFSA) reported that in 2013, 1763 human cases of listeriosis were described in 27 member states^33^. Food vehicles were: crustaceans, shellfish, mollusks, cheese, meat, vegetables, juices and products thereof (mixed salad)^33^.

The increase of the listeriosis outbreaks pushed governments and food industry to take action to drastically reduce this problem^34,35^. Thus, it appears evident that the improvement of existing detection methods is necessary to reduce the cases of consumer illnesses and deaths and their associated economic costs^36^.

For the detection of LM different conventional methods have been developed: plate colony count, biochemical tests, molecular assays, and immunoassays. Nevertheless, these methods may take up to a week to yield a confirmed result. For example, the enrichment step of LM before the detection takes long time. In fact, depending on initial concentration of Listeria from 1 to 7 days are required to see a visible colony on agar or to highlight a bacterial growth in culture media. Molecular and immunological tests reduce the enrichment time to 18 h/72h^37^. In order to improve detection methods of LM, biosensor technology has been developed^38–40^. This approach is based on the interactions between the bacteria and bio-receptors immobilized on different solid-state supports^41^.

However, all the existing detection methods share the same three handling procedures: enrichment of bacterium target, isolation of the pathogen and confirmation of it^23,42^.

The aim of this study was to use a chitosan-cellulose nanocrystal (CNC) membrane-based fluorescence immunoassay to develop a new rapid method for detection of *L. monocytogenes* on surface sampling.

## Materials and methods

### Materials

All the used reagents were chosen with a purity grade ≥ 98.0%. 1-[3-(Dimethylamino)propyl]-3-ethylcarbodiimide (EDC), N-Hydroxysuccinimide, N,N-Dimethylformamide, bovine serum albumin (BSA; fraction V), non-fat dried milk, gelatin from cold-water fish skin, tripolyphosphate (TPP), and 3,5-tetramethylbenzidine (TMB) were acquired from Sigma-Aldrich (St. Louis, MO, USA). Microplates (96-well) Nunc LockWell MaxiSorp C8 form, F16MOD black 96 microplates and the fluorescent probe (AF488) Alexa Fluor 488 NHS Ester (Succinimidyl Ester) purchased from Thermo Fisher SCIENTIFIC (Waltham, Massachusetts, USA). Sephadex G-25 Fine, Polyvinylidene Difluoride (PVDF) transfer membranes 0.45 nm and reagents utilized for Western blot experiments acquired from Amersham Biosciences (GE Healthcare Switzerland). Goat polyclonal to rabbit and mouse IgG—HRP conjugate (secondary antibody) was from abcam (Cambridge, UK). Chitosan 85/1000 (CHI) for pharmaceutical applications was purchased from Heppe Medical Chitosan GmbH (Halle, Germany). FPInnovations (Pointe-Claire, QC, Canada) provided nanocellulose crystals. Tryptic Soy Broth (TSB), Brain Heart Infusion (BHI) and Tryptic Soy Agar (TSA) purchased from Alpha Biosciences (Baltimore, MD, USA). X-ray films were purchased from FUJIFILM Inc. (Minato, Tokyo, Japan).

### Bacterial strains and growth conditions

Five bacterial strains were chosen: *L. monocytogenes* HPB 2812, *L. innocua* (LSPQ 3285), *E. Coli* 0157:H7 EDL933*, E. coli* NP 25922, *S. Typhi* SL1344 all purchased from Laboratoire de Santé Publique du Québec (Ste-Anne-de-Bellevue, QC, Canada). Bacteria were revived from cryovials stored at − 80 °C and spread onto TSA plates for 24 h at 37 °C. The obtained colonies were inoculated in 10 mL TSB, and grown following the previous conditions. Serial dilutions of fresh culture were sub-cultured in 10 mL of TSB and BHI and growth at 37 °C, 120 rpm for 24 h. To compare the medium effects on the bacterial growth was used the BHI that is an enrichment medium with no selective agent^43^. Based on Coutu et al.^24^ as a standard broth was used TSB. For the enumeration of the bacterial cultures, it was performed the plate counting method diluting 1 mL of the sub-culture in 9 mL of saline water prior to spreading 100 mL on Tryptic Soy Agar plates, in triplicate. Colonies were counted on TSA to determine the initial concentration of the cultures. A nutrient broth containing 10^2^ CFU/mL of *L. monocytogenes* HPB 2812 bacteria was growth for 24 h, and the growth was monitoring each hour from 1 to 8 h and at 24th hour. *L. monocytogenes* HPB 2812 (serotypes 1/2a) was used for LM detection by sandwich ELISA test. The other strains were used for monitoring the presence of cross-reactivity.

### Antibodies and recombinant p60 protein production

Recombinant protein p60 was produced and purified by GenScript USA Inc. (NJ, USA)^23^. Briefly, competent *E. coli* BL21 were transformed with pUC57-p60rec DNA plasmid. The produced p60 protein was purified by affinity chromatography (Ni–NTA agarose matrix) via polyhistidine-tag.

The obtained p60 protein was utilised to produce the polyclonal antibodies anti-p60 protein (LM pAb), which also carried out by GenScript USA Inc. The specificity and the titer of the purified antibodies were screened using ELISA and Western Blot.

Mouse monoclonal antibodies anti-p60 pepD (LM mAb) were produced according to Beauchamp et al.^23^ and Coutu et al.^24^. The antibodies recognize a short unique peptide D (PepD) composed of 11 amino acids (QQQTAPKAPTE) of the *L. monocytogenes* p60 protein. The purified antibodies were purchased from AdipoGen LIFE SCIENCES international Inc. (Cedarlane, Burlington, ON, Canada).

### SDS-PAGE electrophoresis and western blot experiments

Western blot experiments were performed according to Varriale et al.^44^. In brief, aliquots of 15 mL of *L. monocytogenes* bacterial cultures growth with two different medium (TSB and BHI) and at different time of growth (1 h to 8 h, and 24 h), were heated at 95 °C in the presence of Laemmli buffer^45^ and were separated on a sodium dodecyl sulphate polyacrylamide gel electrophoresis (12% SDS-PAGE). After separation, the proteins were transferred onto a PVDF membrane with Bio-Rad TRANS-Blot turbo. The obtained membranes were blocked for 1 h at room temperature with 50 mL of the blocking buffer (TBS supplied with 5% non-fat dried milk). After three washing steps with TBS-T (10 min for each washing), the membrane filters were incubated with a rabbit polyclonal (LM pAb) and mouse monoclonal (LM mAb) antibodies against the p60 protein (dilution 1:2000 in the diluting buffer) for 1 h at room temperature. The same procedure was performed with secondary antibodies, goat anti-rabbit and anti-mouse HRP conjugate (diluted 1:6000 in the blocking buffer) respectively. After three washing steps, proteins were visualized by chemo-luminescence using the Amersham ECL plus and X-ray films developed manually in the darkroom. A recombinant p60 protein was used as a positive control.

### Fluorescence labelling of polyclonal IgG anti-p60 protein (LM pAb)

LM pAb were labelled with the fluorescent probe, Alexa Fluor 488 NHS ester (AF488). The labelling was achieved according to the supplier instructions. In brief, 0.1 mL of an antibody solution (7.5 mg/mL, 20 mM PBS pH 7.4) was mixed with 5 mL of AF488 (10 mg/mL) and 12 mL of 1 M sodium bicarbonate pH 8.3. The molar ratio of the dye and the protein were kept 12:1; the reaction was performed 1 h at 25 °C under stir. LM pAbs AF488 labelled (LMpAb488) were purified from the unreacted probe by a Sephadex G-25 column. The labelled IgG collected were pooled and were concentrated to a final volume of 2.0 mL by 10 kDa micro-concentrator (Sartorius Vivaspin 500 Polyethersulfone). To be sure that the entire unreacted probe was eliminated from the antibody solution, an extensive dialysis procedure was performed against 20 mM sodium phosphate 150 mM NaCl pH 7.4 by using dialysis tubes with a cutoff of 3500 Da (Spectrum Labs). The degree of labelling (DOL) calculated, following the supplier instructions, was found to be 6.0.

### Steady-state fluorescent measurements

The fluorescence emission of LMpAb488 was monitored on Infinite M1000 Tecan microplate fluorimeter. To excite selectively AF488, the excitation wavelength was fixed at 495 nm (Ex. bandwidth at 2.5 nm; emission bandwidth at 5 nm). Emission spectra were acquired from 500 to 650 nm, at 1.0 nm intervals, at a scan speed of 100 nm/min using a 96 multiwell plate F16MOD black (Thermo Fisher SCIENTIFIC). All measurements were carried out at 25 °C (PBS buffer pH 7.4, 200 ml). The buffer alone was used as blank and its emission contribution was subtracted from the experimental spectra. LMpAb488 concentration determined from absorbance value at 278 nm using the extinction coefficient equal to 210000 mol^−1^ cm^−1^; to prevent the inner filter effect^46,47^ all measurements were performed on a sample with an optical density at 295 nm less than 0.1OD. All measurements were performed in triplicate, the values acquired were averaged, and the blank was subtracted and normalized by the maximum. All data obtained were analyzed and plotted using OriginLab 8.0 software.

### Preparation of the chitosan-cellulose nanocrystal CNC membrane

Etty et al.^48^ optimized CNC membrane containing chitosan, nanocrystal cellulose and glycerol. Nanocrystal cellulose powder was dissolved in water (0.6% w/v) at 25 °C, under vigorous stirring for 16 h. After homogenization (T 25 digital ULTRA-TURRAX Disperser), the obtained solution of nanocrystal cellulose was mix with 1% (v/v) of glacial acetic acid. Following, the viscous was solution was treated with QSonica Sonicator for 1000 J/grams of nanocrystal cellulose. Afterwards, 2% (w/v) of chitosan powder was added under vigorous stirring at room temperature, for 4 h. After these 2 h, 0.6% of glycerol (v/v) was added at the blend and the suspension obtained was mixed again for 1 h for total dissolution. The polymer blend obtained was degassed under vacuum for 16 h. Finally, 1 g of the chitosan-cellulose nanocrystal suspension degassed and cast in each well of F16MOD black 96 microplates. The drying of the CNC suspension in the microplate was done at 25 °C and under vacuum (7 days) in the aim to obtain the completely dry CNC membrane. The dried membranes were preserved at 25 °C.

### Optimization of the LM mAb immobilization onto the CNC membrane

Three different immobilization protocols on CNC membrane 96-wells microplate were been tested and compared: (1) glutaraldehyde chemistry (TPP + GA), (2) Carbodiimide chemistry (TPP + EDC/NHS), (3) Carbodiimide chemistry (EDC/NHS).

The protocol (1) and protocol (2) required as follow: the CNC membrane was activated with 300 mL of sodium tripolyphosphate (TPP) 5% (w/v) and the reaction was conducted at 25 °C, 80 rpm for 10 min. In addition, to remove the excess of TPP, five washing steps were performed (10 min each washing step) with 300 mL of distilled water.

The TPP step was not performed when protocol (3) was used.

Then, for the protocol (1), the amino groups of the CNC membrane were bound to the aldehyde groups of glutaraldehyde (300 mL of GA, 0.2% (v/v)) during 1 h at 25 °C, 80 rpm. Thereafter, three washing steps with 300 mL of distilled water were performed. Subsequently, 100 mL of the LM mAb solution (2, 5 and 10 mg/mL) was linked to the free aldehyde group added on the CNC membrane. This second crosslinking reaction was conducted for 2 h, at 25 °C and 80 rpm. Then, the wells were rinsed three times for 10 min and two times for 5 min with PBS-T buffer under shaking, to remove all the unbound antibodies. As regard the protocol (2), the LM mAb solution (2, 5 and 10 mg/mL) was incubated with a mixture of EDC/NHS (0.4/0.1 M) for 15 min and subsequently incubated in the wells 2 h at 25 °C and 80 rpm. After the crosslinking antibodies immobilization were performed three washing of 10 min and two washing of 5 min with PBS buffer supplied in presence of 0.05% (v/v) Tween 20 (PBS-T), and the films were kept under shaking to remove the unbound antibodies. For protocol (3) was applied the same protocol followed for protocol (2) unless the TPP step.

To evaluate the performance of the three different immobilization methods (TPP + GA, TPP + EDC/NHS and EDC/NHS chemistry), indirect ELISA experiments have been performed. Thereafter, to block non-specific binding on the CNC membrane with immobilized LM mAb, 300 mL of three different blocking solution (bovine serum albumin 1% (w/v) in PBS, nonfat dried milk 5% (w/v) in PBS and Fish gelatin 1% (w/v) in PBS) has been tested and compared. The wells were incubated at 25 °C, 1 h and 80 rpm followed by three washing with 300 mL of PBS-Tween.

20 (PBS-T) for 10 min. After that, the wells were incubated (1 h at 37 °C) in the presence of 100 mL of HRP anti-rabbit antibodies, (1/6000). After three washing steps with PBS-T, the evaluation step was done by adding of 3,3′,5,5′-tetramethylbenzidine (TMB) substrate (10 min at room temperature), and H_2_SO_4_ 2 M (v/v) was added (to stop the enzymatic reaction) on the microplate. Samples were read at 450 nm using a microplate reader BioTek ELx800 equipped with Gen5 software.

### Detection of *L. monocytogenes* by fluorescent ELISA sandwich assay

To detect LM*,* the optimized 96 wells microplate covered with a CNC membrane was used. Following the protocol (2) a volume of 100 mL (10 mg/mL) of the LM mAb were immobilized. Thereafter, 300 mL/well of a PBS buffer containing 5% (w/v) of no-fat dried milk was used to block the unspecific interaction (1 h at 25 °C). Then, the wells were washed three times (10 min each) with 300 mL of PBS with 0.05% Tween 20 (PBS-T). After the washing, 300 mL of bacterial culture sample (previously described) was added in each well, incubation was performed for 1 h at 37 °C. Then, three washing steps of 10 min each, with PBS-T were performed. Thereafter, 100 mL/well of detection antibodies LMpAb488 (10 mg/mL in PBS-T supplied with 1%, no-fat dried milk) added onto the microplate and incubated 1 h at 37 °C. Successively, three washing steps were done to remove all unbound detection antibodies. Revelation step was done directly by reading the fluorescence emission on Infinite M1000 Tecan microplate fluorimeter. To excite selectively AF488, the excitation wavelength was fix at 495 nm. (Ex. bandwidth at 2.5 nm and emission bandwidth at 5 nm). Emission spectra were acquired from 500 to 650 nm, at 1.0 nm intervals, at a scan speed of 100 nm/min. All measurements were carried out at room temperature in PBS buffer pH 7.4.

### Sampling on simulated bench-working surface

**Figure 1:**
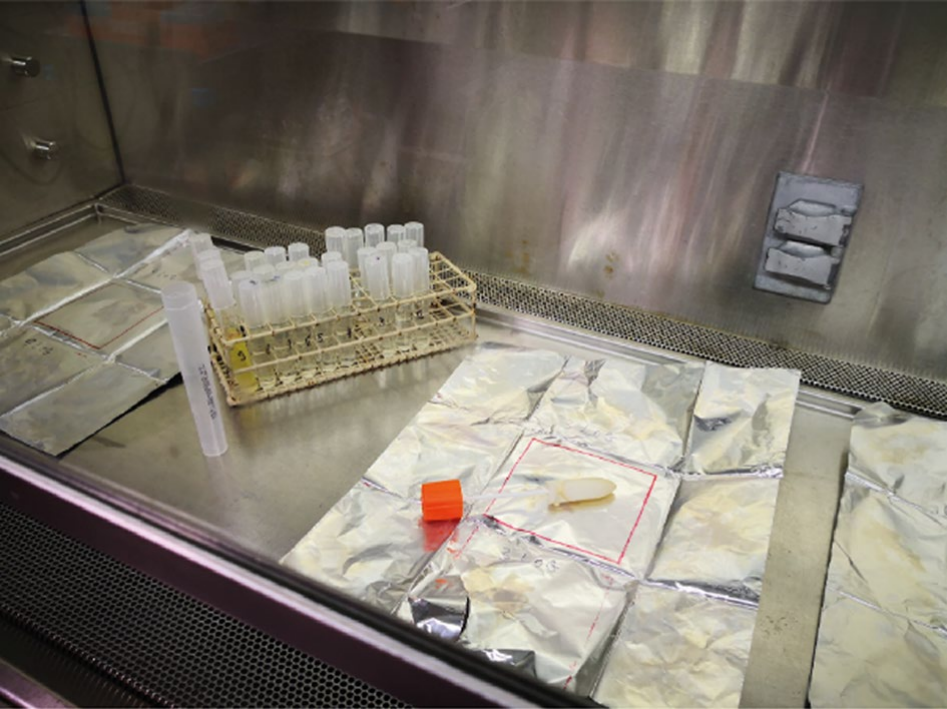
Sampling procedure performed on simulated bench working surface. Sampling procedure performed on simulated bench working surface (alumina foil). The alumina foil was been contaminated with LM cultures, following the procedure described in the “Materials and methods” section.

Serial dilutions (to 9 Log from 1 Log) of 1 mL of a sub-culture of bacteria in TSB were performed in 9 mL of the 0.1% peptone water (w/v) prior to spreading (100 mL) in triplicate onto PALCAM agar plates (37 °C for 48 h). The number of vital bacteria (CFU/mL) in the sub-culture was determined from the colonies grown on PALCAM agar plate. A quantity of 100 mL of each bacteria dilutions was spread on a sterile surface of 900 cm^2^ (30 cm × 30 cm) of aluminium foil (Fig. 1), according to Canada procedures for food and environmental samples^49,50^. A swabbing of the contaminated surface was performed with the two faces of dry sponge-stick (3 M, ON, Canada) by “Z” patterns illustrated in Canada public health methods (Canadian Food Inspection Agency (ACIA) 2013). The contaminated sponge-sticks were incubated in 10 mL of TSB medium and growth for 8 h at 37 °C. Cultures obtained were used to detect LM by optimized fluorescent sandwich immunoassay on the CNC membrane. The cross-reactivity was evaluated by the same procedure on five different bacterial strains.

### Statistical analysis

All measurements were done in triplicates and the value reported is the arithmetical mean subtracted by the blank and normalized by the maximum. For each sample (different culture or different dilution points), three replicates were analyzed. For each culture, several dilution points were collected (Log 9, Log 8, etc.). The mean and standard deviation (SD), of each sample, were calculated. Each measurement reports the standard deviation. The data analysis was performed by Origin 8 Pro software. The limits of blank (LOB) and of detection (LOD) of the assay were estimated according to Armbruster et al.^51^.

## Results and discussion

LM is a foodborne pathogen that causes human listeriosis.

In this work we have combined a CNC membrane support with the specificity and binding capability of the antibodies to the sensitivity of the fluorescence molecules to realize an immunoassay to detect the presence of LM on simulated bench-working surface.

Our biosensor platform is a fluorescence sandwich enzyme-linked immunosorbent assay (FSELISA) for surface sampling based on chitosan and nanocrystal cellulose biopolymer. The ELISA multi-well microplates were covered with a CNC membrane. Chitosan [α (1–4) 2-amino-2-deoxy β-D-glucan] was chosen as the principal component of the membrane due to its film-forming properties and the presence of free amino groups available for antibody immobilization^52^. Nanocrystal cellulose and chitosan were used as filling agents because they have reinforcing properties in polymer matrices such as water-soluble and water-insoluble polymers to produce reinforced nanocomposites^53–56^.

With the aim to improve the antigen capture during bacterial growth and reduce the time of analysis, the anti-PepD monoclonal capture antibodies were covalently immobilized on the CNC membrane by ethyl-(dimethyl aminopropyl)carbodiimide (EDC) and *N*-hydroxysuccinimide (NHS) coupling chemistry. The anti-p60 protein polyclonal detection antibodies were labelled with the fluorescence probe Alexa Fluor 488. The presence of LM on simulating food industry surfaces was evaluated by acquiring directly the fluorescence signal by the microplate reader fluorometer.

The immobilization of LM Ab onto a CNC membrane allowed for an improvement of the p60 protein-specific antigen-capture that reduces the time of LM growth needed as the enrichment step. The time reduction of the enrichment step in combination with the use of the fluorescence detection sensitivity allowed to perform LM detection in 12 h instead of 18–24 h compared to the conventional methods. Based on previous results obtained from our group^23,24,57^, we developed an assay that exhibits a LOD of 10^2^ CFU/mL and a better specificity compared with others five most diffused pathogenic bacterial strains.

### Bacterial growth and p60 protein expression evaluation

**Figure 2:**
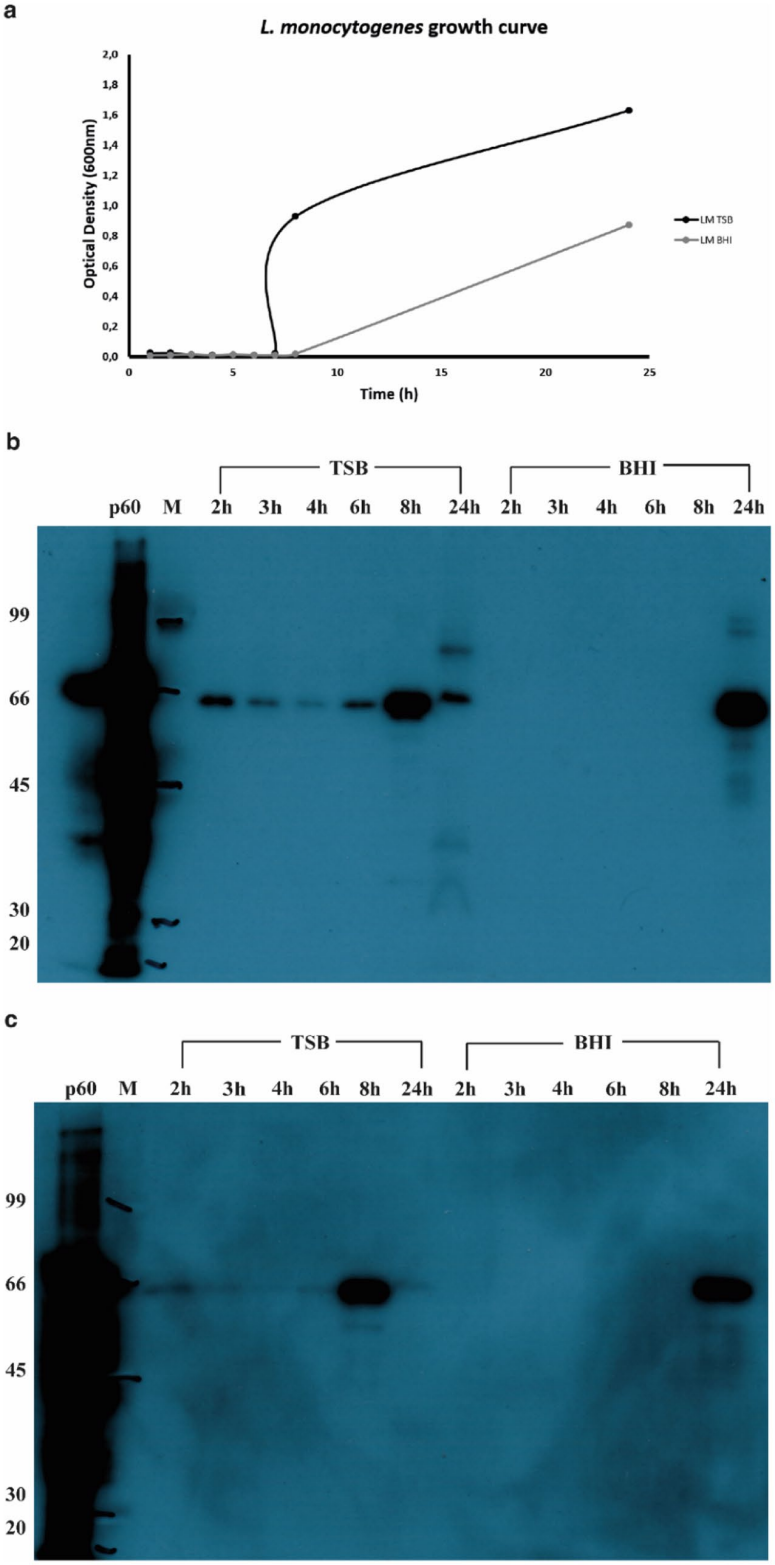
Bacterial growth curve and Western-blot analysis of the p60 production. **(a)** The kinetic bacterial growth curves—black colour—the results obtained with the TSB medium; grey colour—the results obtained with the BHI medium and **(b)** Western-blot with the LM pAb and **(c)** Western-blot with the LM mAb. p60 is the recombinant protein; M: molecular size marker; 2 h, 3 h, 4 h, 6 h, 8 h and 24 h was the sample collected during the growth grouped by culture medium used (TSB and BHI).

10 mL of (10^2^ CFU/mL) LM HPB 2812 culture, prepared as described in “Bacterial strains and growth conditions” under “Materials and methods” section, was growth in TSB and BHI medium up to 24 h, at 37 °C under shacking (120 rpm). The OD of the growth has been monitored each hour from 1st to 8th, and at the end after 24hth. The kinetics curve (Fig. 2a) of LM growth was evaluated by three parameters: the maximum asymptotic value of growth (A24h), the Lag phase duration, and the maximum specific growth rate of Log phase (Table 1). In accordance with the results reported by Etty et al.^23,58^, the best growth was achieved with TSB, with an A24h of 1.63 OD compared with 0.87 OD obtained for the BHI medium. The Lag phase in TSB (l =  ~ 6 h) was shorter than BHI (l =  ~ 8 h) and the same results were achieved for the maximum specific growth rate of Log phase, m_m_ = 0.166 (h^−1^) and m_m_ = 0.125 (h^−1^) respectively for TSB and BHI. These results suggested that the amount of p60 protein produced by LM was higher when the bacteria was growth in the TSB medium than the BHI.

**Table 1:** Parameters of growth for LM in two media.

Medium	A_24h_	m_m_ (h^−1^)	l (h)
TSB	1.63	0.166	6.0
BHI	0.87	0.125	7.0

*TSB* tryptic soy broth; *BHI* brain heart infusion; A_24h_ is the maximum asymptotic value of growth after 24 h of incubation; m_m_ is the maximum specific growth rate of log phase (m_m_ = 1/l). l is the lag time.

To verify our hypothesis, the amount of p60 protein produced and released in the culture medium by LM has been evaluated by western-blot experiments.

The samples collected each hour from the 1st to 8th and at 24th were tested with both LM pAb and LM mAb, as shown in the Fig. 2b,c respectively. Both the used antibodies recognized the recombinant p60 protein and the 66 kDa characteristic band of the p60 bacterial protein. In accordance with our hypothesis, it was possible to detect the p60 protein after only 2 h from the TSB culture; otherwise, from the BHI culture during the first 8 h of growth was not still possible to detect the presence of the p60 protein. Based on these results we focused our study on LM growth in the TSB medium.

### Labelling of polyclonal IgG anti-p60 protein (LM pAb)

**Figure 3:**
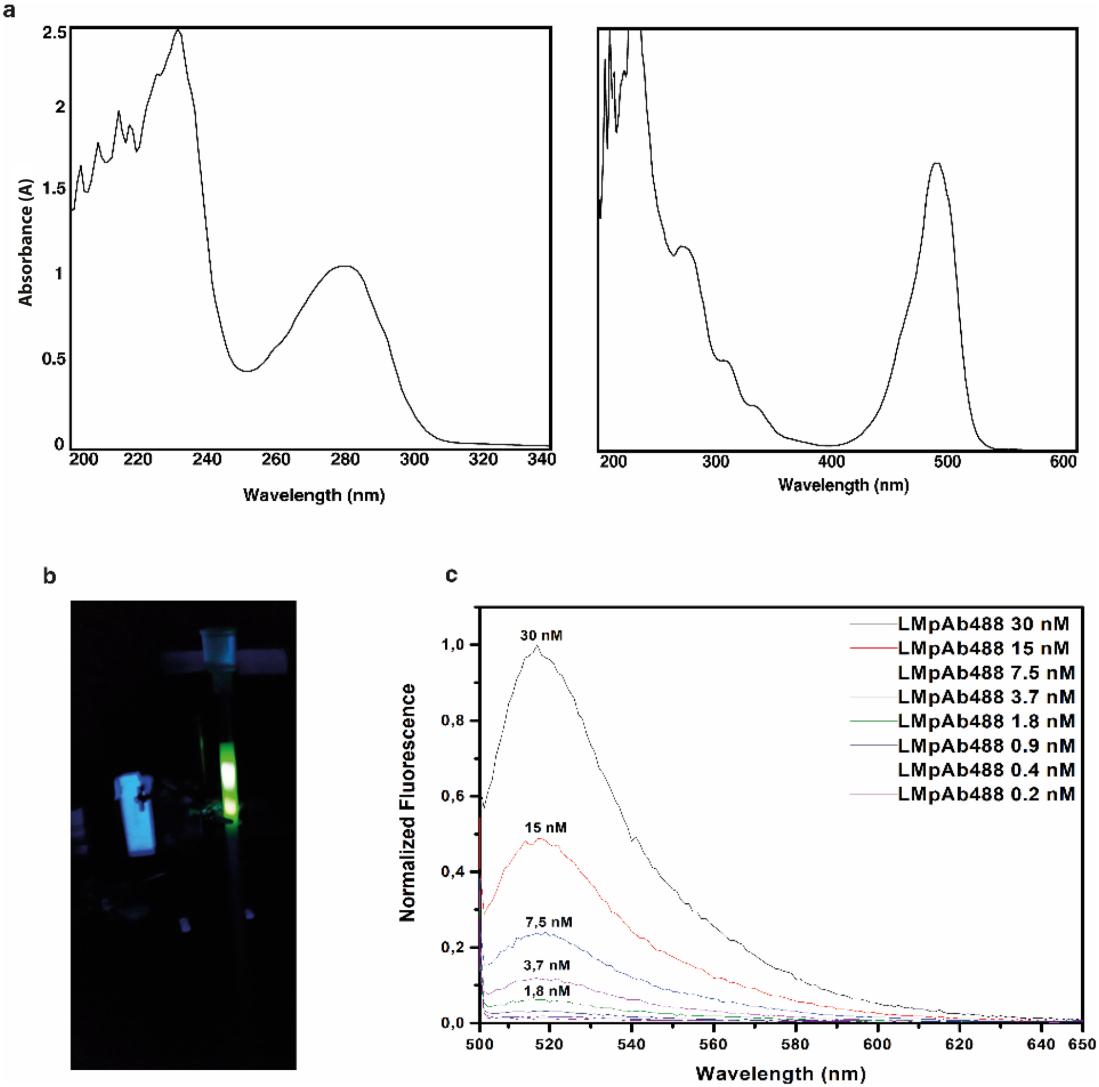
Labelling of the LM pAb with Alexa Fluor 488 dye and its fluorescence emission analysis. **(a)** Absorbance of LM pAb -on the left- and LMpAb488 -on the right-. After the labelling procedure, appeared an absorbance peak at 495 nm that confirmed the presence of dye bound to the antibody. **(b)** Exclusion chromatography phase: two peaks are present, -bottom- labelled antibody fraction and -top- the unreacted probe. **(c)** Normalized fluorescence emission spectra of LMpAb488 excited at 495 nm. The emission peak centered at 520 nm.

A quantity of 750 mg of purified LM pAb (Fig. 3a) was mixed with 50 mg of Alexa Fluor 488 NHS ester to respect the molar ratio dye/protein of 12:1. The amide-bond reaction has been conducted in 1 M bicarbonate pH 8.3 buffer to drive the dye to bond on the lysine residues present on the antibody surface. After 1 h of incubation, the reaction was stopped and the unreacted dye has been removed by a size exclusion chromatography. Figure 3b shows a step of the chromatography, followed by an UV-lamp where it is clear that the unreacted dye (upper spot) has been divided from the labelled antibodies (bottom spot). Based on the shape and the size, the dye runs slower than the labelled antibodies that have been collected before. The collected fractions of labelled antibodies were evaluated by spectrophotometry and pooled (Fig. 3a). The efficiency of the labelling procedure was confirmed by the analysis of the labelled antibodies absorbance spectra, the co-presence of two peaks, one of the antibodies (centered at 278 nm) and the other one of the dyes (center at 495 nm) (Fig. 3a). After several dialysis steps, the calculated DOL of the labelling reaction was 6.0; the yield after the labelling reaction and the purification was 450 mg concentrated at 4.5 mg/mL (30 mM).

In order to explore the instrumental sensitivity and understand the LMpAb488 concentration useful for the immunoassay, a preliminary test was performed. The LMpAb488 stock solution was diluted 1000 time and then an eight-point serial dilution (1:2), from 4.5 mg/mL (30 nM) to 0.035 mg/mL (0.2 nM), has been tested. A volume of 200 ml/well of bacteria culture, for each point of the serial dilution, was spotted on F16MOD black 96 microplates. Figure 3c shows the normalized fluorescence emission of the purified LMpAb488 evaluated by Infinite M1000 Tecan microplate fluorimeter. A good signal was been observed up to 0.14 mg/mL.

### Monoclonal antibody (LM mAb) immobilization onto the CNC membrane

**Figure 4:**
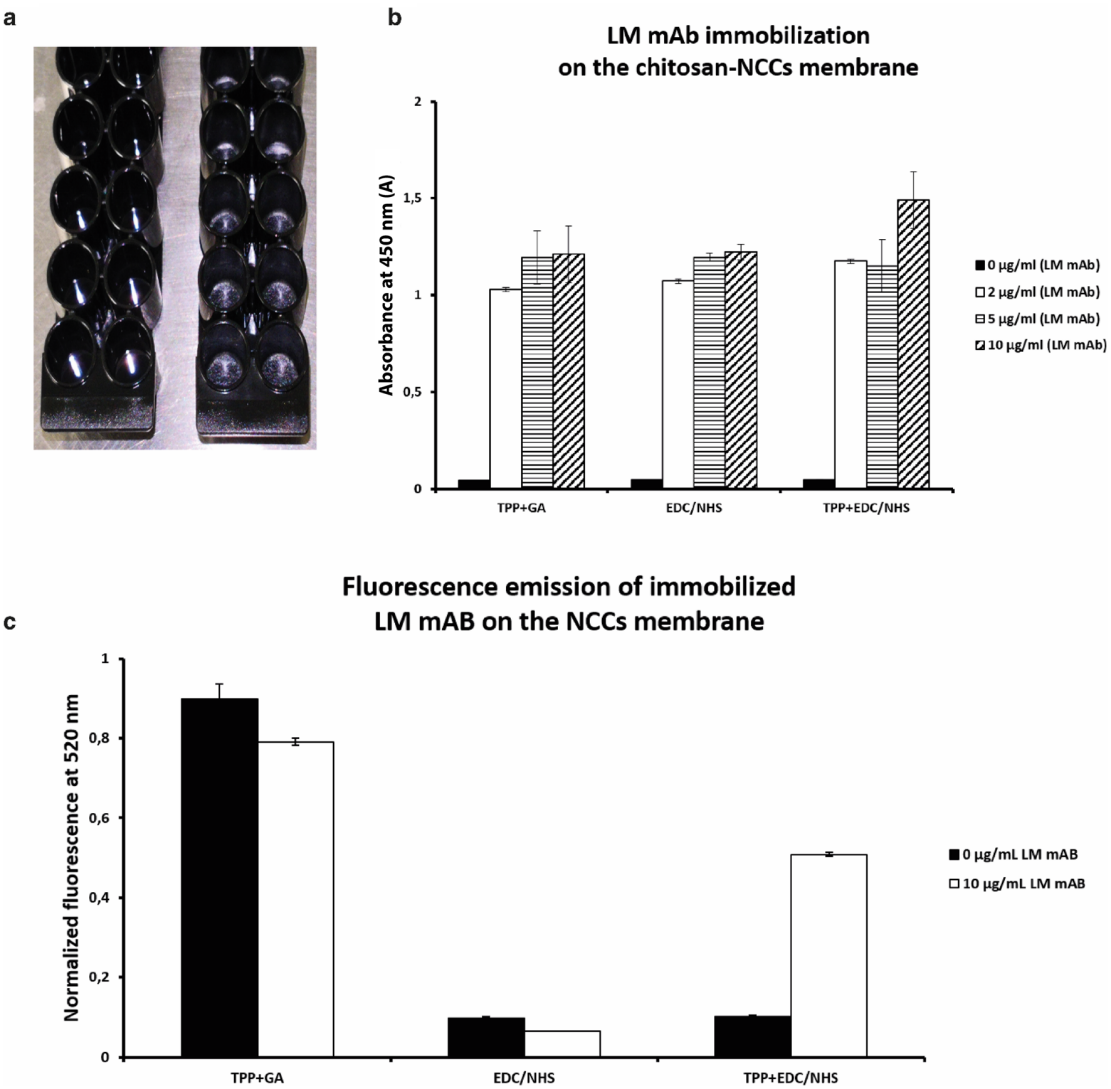
Multiwell microplate preparation: CNC membrane deposition and immobilization of capture antibody. **(a)** F16MOD microplate details: left side—clean wells and right side—CNC membrane coated wells. **(b)** Absorbance emission at 450 nm for the different LM mAb immobilization protocols performed. The best immobilization was been obtained at 10 mg/mL of monoclonal IgG, following protocol number two. **(c)** Normalized background fluorescence emission of each performed protocols.

A quantity of 1gr of CNC membrane degassed suspension was dispensed in each well of the F16MOD black 96 microplates and dried under vacuum for 7 days (Fig. 4a). Respective quantities of 2, 5 and 10 mg/mL of LM mAb were covalently immobilized, as capture element, on the CNC membrane following three different protocols: TPP + GA (glutaraldehyde chemistry), TPP + EDC/NHS chemistry and EDC/NHS chemistry without TPP. To evaluate and compared the results of the three different immobilization protocols, an indirect ELISA experiment was performed. Three different blocking solutions were tested: 1% gelatin, 1% serum bovine albumin and 5% dried nonfat-milk. The best blocking solutions resulted in the 5% dried nonfat-milk (data not shown).

Figure 4b shows that the best LM mAb value of the immobilization on the CNC membrane (1.5 Abs) was achieved performing the TPP + EDC/NHS protocol with a mAb concentration of 10 mg/mL. Lower immobilization level was achieved with the other two protocols performed at each tested mAb concentration of 2, 5 and 10 mg/mL. As reported by Etty et al.^48,58^ the insolubilizing pretreatment of the chitosan membrane with TPP improves the efficiency of the immobilization protocol and reduce the deformation and brittleness of the support improving the mechanical properties of the membrane.

To develop and to optimize the fluorescence sandwich immunoassay, the background fluorescence emission of the immobilized antibodies was evaluated. In Fig. 4c, the graph reports the normalized background fluorescence emission of each performed protocols. The fluorescence emission of the glutaraldehyde protocol turns out eight-time greater than the other two protocols. Shin et al.^59^ described in details the glutaraldehyde auto-fluorescence emission in the interaction between the chitosan and some polymers cross-linked by glutaraldehyde. Fluorescent Schiff base bonds (C=N) and double bonds (C=C) were generated simultaneously by crosslinking of the amine moiety of the cationic polyelectrolytes with monomeric glutaraldehyde or with polymeric glutaraldehyde. The acquired results represent an added value that strengths protocol (2) as the best choice for the optimization of the capture antibody immobilization protocol. To develop a fluorescence sandwich assay, it is relevant to maintain as low as possible the signal/noise ratio.

For this reason, to immobilize the antibodies on the chitosan membrane, it was performed the TPP + EDC/NHS chemistry protocol that resulted to be the most efficient protocol among all the tested protocols.

### Optimization of the fluorescent sandwich immunoassay

**Figure 5:**
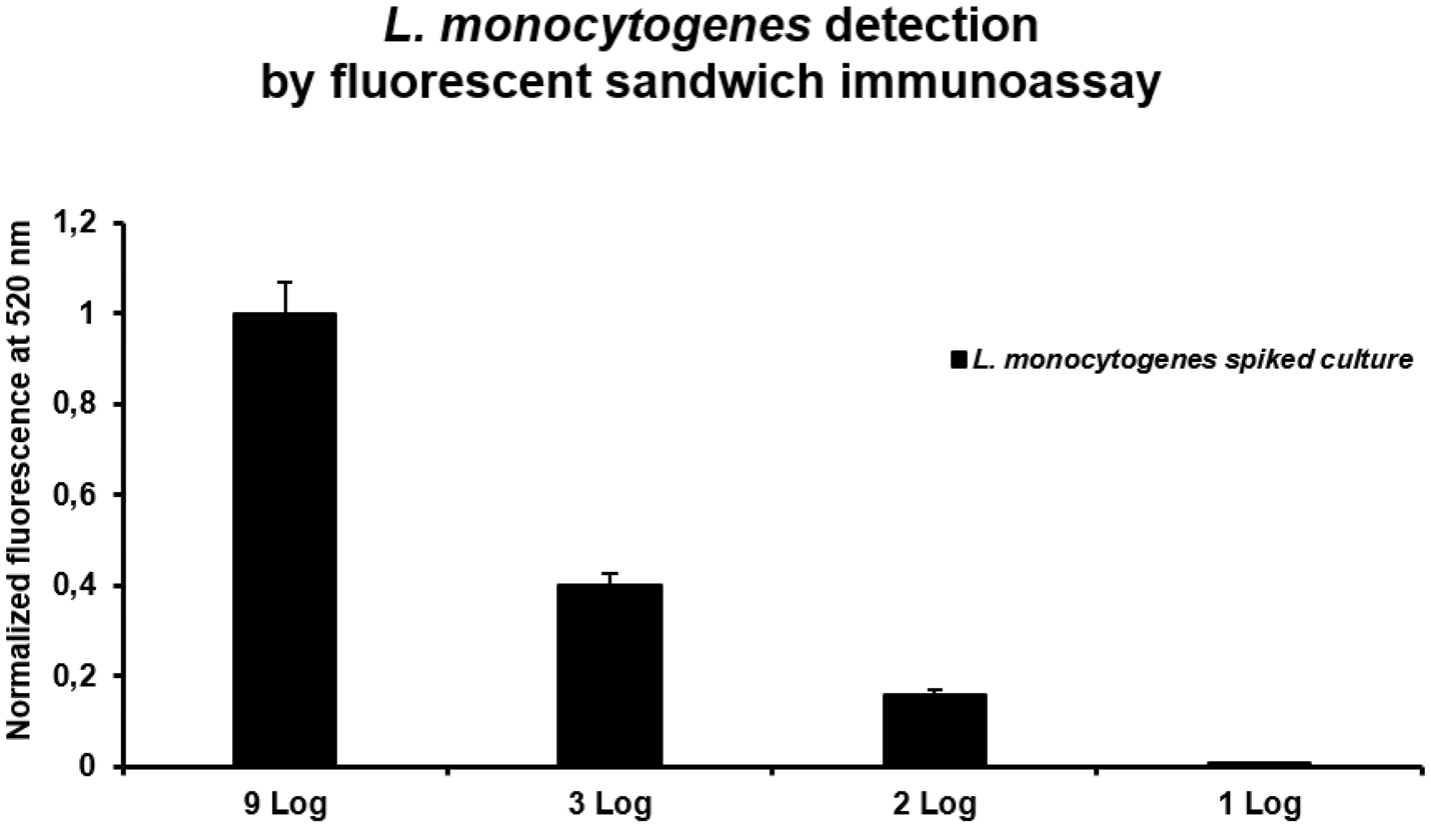
Sandwich fluorescence immunoassay: spike test. Sandwich fluorescence immunoassay of LM spiked samples (8 h of growth).

After the optimization of the capture antibody immobilization on the CNC membrane, to develop the fluorescent sandwich immunoassay, the optimization of the detection antibody was been required. Three concentrations of LMpAb488 (2, 5 and 10 mg/mL) were tested, and the best result was obtained with 10 mg/mL (data not shown). Figure 5 reports the results for several spiked samples of LM culture after 8 h of growth in TSB medium. The samples were prepared from different concentrations (9, 3, 2, and 1 Log) of LM cultures, and verified by plate counting method. A volume of 100 mL of each sample was spotted in the well covered with the CNC membrane modified with the LM mAb covalently bound. After 1 h of incubation at 37 °C, the bacteria culture was removed and TBS-T buffer was washed the unbound bacteria. After that, 100 mL of 10 mg/mL LMpAb488 were added in each well and incubated 1 h at 37 °C. After, another washing step was performed (10 min) with 100 mL of TBS buffer. The fluorescence emission was acquired by the Infinite M1000 Tecan microplate fluorimeter. The maximum signal reached at 10^9^ CFU/mL culture was 1.00 and the limit of detection (LOD) was at 10^2^ CFU/mL (0.16) while the limit of the blank (LOB) was 0.074.

### *L. monocytogenes* detection on the simulated bench-working surface

The developed immunoassay was performed to detect the presence of LM on the simulated bench-working surface. In brief, 100 uL of each five different bacteria culture, in three different concentrations (9, 3, 2, and 1 Log) were spread on the sterile aluminium foil (simulated surface). After that, swabbing of the dry-contaminated surface was performed with the two faces of dry sponge-stick and the sponge was immersed in 10 mL of TSB and incubate 8 h at 37 °C.

**Figure 6:**
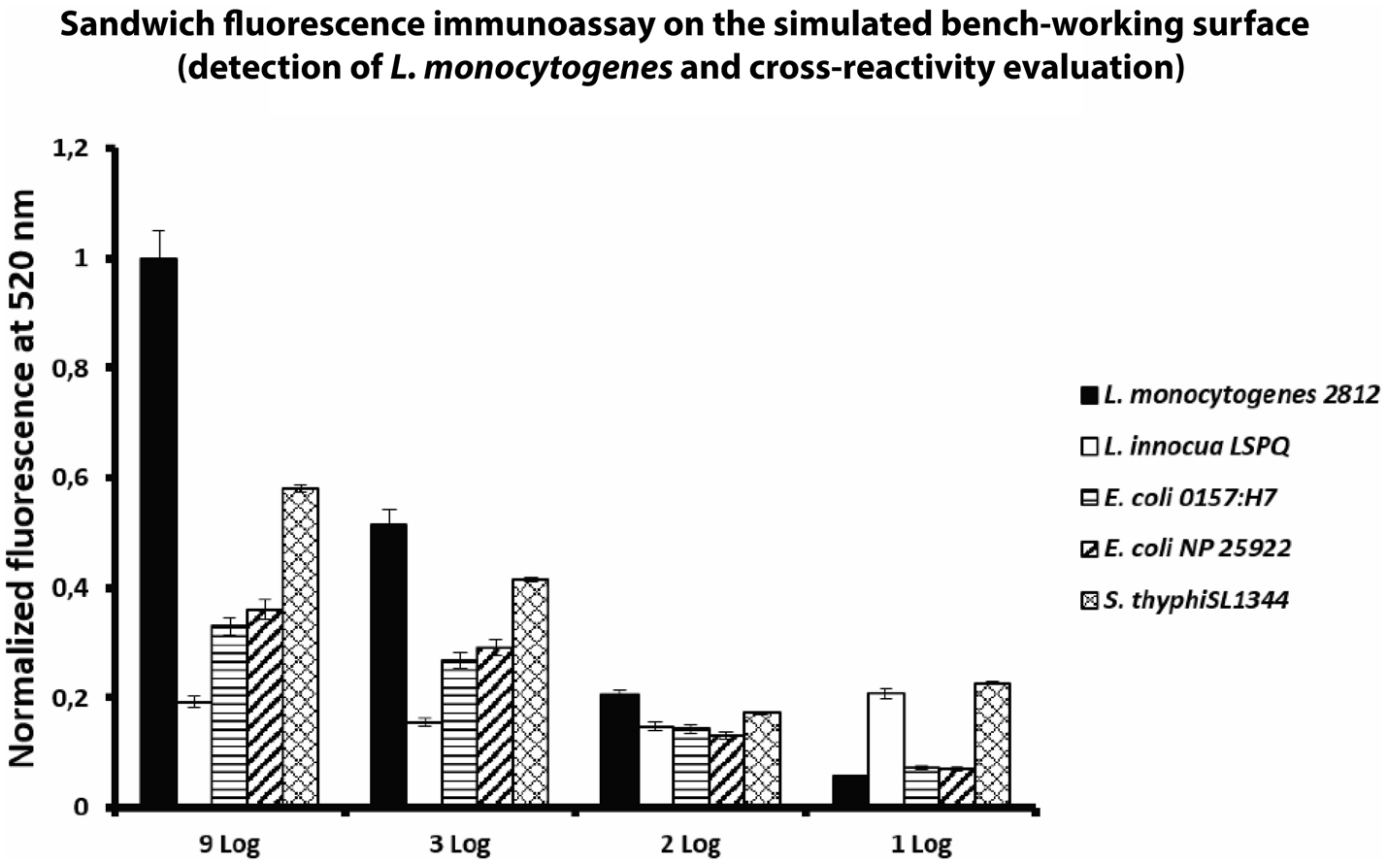
Sandwich fluorescence immunoassay on the simulated bench-working surface (detection of LM and cross-reactivity evaluation). Sandwich fluorescence immunoassay: four dilutions were been tested (9 Log, 3 Log, 2 Log and 1 Log) of five different bacteria strains. The developed assay shows a LOD value of 2 Log.

After the growth, the immunoassay was performed as previously described in the “Materials and methods”, “Detection of L. monocytogenes by fluorescent ELISA sandwich assay”. Figure 6 reports the results obtained for the detection of LM from the contaminated simulated surface. Comparing the fluorescence emission values between the tests performed with the spiked samples reported in Fig. 5, there is a slight difference in the fluorescence emission values probably due to the biological difference of the bacteria culture and the operator in the sampling procedure. Despite in the Fig. 6 is reported a low signal for 1 Log dilution (0.056), the limit of detection (LOD) is fixed at 10^2^ CFU/mL (0.2) since the limit of the blank (LOB) was the same at 0.075.

The developed assay shows the ability to detect with high specificity LM because for each bacteria concentration tested was able to distinguish the presence of LM from the other bacteria strains tested. The graph in Fig. 6 shows a lower fluorescence emission for the others bacteria strains tested. However, these fluorescence emission values are lower than the fluorescence emission values recorded for the LM. Consequently, it is possible to have a differential analysis. In terms of sensitivity, performing the assay on the simulated bench-working surface, the limit of detection was preserved.

## Conclusion

In this work is reported the development of a novel fluorescent immunoassay to detect the presence of the *L. monocytogenes* on the simulated bench-working surfaces. The developed fluorescent sandwich immunoassay is based on the covalent immobilization of the LM mAb (by an amide bond) on the CNC membrane. The optimized TPP-EDC/NHS immobilization protocol of the capture antibody on the CNC membrane, permits to improve the p60 protein-specific antigen binding capability compared to the conventional support of sandwich immunoassay.

Furthermore, the labelling with the Alexa Fluor 488 of the LM pAb used as detection elements, improves the sensitivity of the assay reducing the time of the analysis because reduce the time of growth needs to detect the presence of LM and guarantee the specificity.

The results show that the developed sensing platform is able to detect the presence of LM after only 8 h of growth/enrichment step and starting from a bacteria concentration of 10^2^ CFU/mL.

This study also showed a reduction of detection time from 18–24 h to 12 h as compared to conventional methods.
